# Crystal structure of an ASCH protein from *Zymomonas mobilis* and its ribonuclease activity specific for single-stranded RNA

**DOI:** 10.1038/s41598-017-12186-w

**Published:** 2017-09-26

**Authors:** Bo-Na Kim, Minsang Shin, Sung Chul Ha, Suk-Youl Park, Pil-Won Seo, Andreas Hofmann, Jeong-Sun Kim

**Affiliations:** 1 Department of Chemistry, Chonnam National University, Gwangju, 61186 Republic of Korea; 2 College of Medicine, Kyungpook National University, Daegu, 41940 Republic of Korea; 3 Pohang Accelerator Laboratory, Pohang, Gyeongbuk 37673 Republic of Korea; 4 Griffith Institute for Drug Discovery, Griffith University, Nathan, Queensland 4111 Australia; 5 Faculty of Veterinary and Agricultural Sciences, The University of Melbourne, Parkville, Victoria 3010 Australia

## Abstract

Activating signal cointegrator-1 homology (ASCH) domains were initially reported in human as a part of the ASC-1 transcriptional regulator, a component of a putative RNA-interacting protein complex; their presence has now been confirmed in a wide range of organisms. Here, we have determined the trigonal and monoclinic crystal structures of an ASCH domain-containing protein from *Zymomonas mobilis* (*Zm*ASCH), and analyzed the structural determinants of its nucleic acid processing activity. The protein has a central β-barrel structure with several nearby α-helices. Positively charged surface patches form a cleft that runs through the pocket formed between the β-barrel and the surrounding α-helices. We further demonstrate by means of *in vitro* assays that *Zm*ASCH binds nucleic acids, and degrades single-stranded RNAs in a magnesium ion-dependent manner with a cleavage preference for the phosphodiester bond between the pyrimidine and adenine nucleotides. *Zm*ASCH also removes a nucleotide at the 5′-end. Mutagenesis studies, guided by molecular dynamics simulations, confirmed that three residues (Tyr47, Lys53, and Ser128) situated in the cleft contribute to nucleic acid-binding and RNA cleavage activities. These structural and biochemical studies imply that prokaryotic ASCH may function to control the cellular RNA amount.

## Introduction

Human activating signal cointegrator-1 (ASC-1) is a transcriptional regulator and a component of a putative RNA-interacting protein complex^1,2^. A prominent feature of ASC-1 is the ASC-1 homology (ASCH) domain (~110 amino acids) situated at the C-terminus^3^, inferred to be involved in RNA processing, and probably conferring RNA-binding properties^1,2^. Proteins containing ASCH domains are widely dispersed across all domains of life, from eukaryotes to prokaryotes^3^. These ASCH domains can be classified into ten families (called 1–10), mostly based on their origins. Most of the available structures of ASCH domains exhibit a central six-stranded β-barrel, that is surrounded by a few α-helices (PDB IDs: 3s9x and 2e5o). However, the biological and molecular functions of ASCH remain ambiguous, largely because no experimental studies have been reported on ASCH domain proteins to date.

The sequence identity between members of different ASCH families is relatively low and sometimes less than 15%^3^. As such, these domains are an example of proteins that have no considerable sequence identity, but nonetheless significant structural similarities^4–7^. In such cases, where amino acid sequence comparison alone fails to provide the required information, comparison of three-dimensional protein structures can deliver fundamental insights to aid the prediction of protein functions. For example, the ‘pseudouridine synthase and archaeosine transglycosylase’ (PUA) domain is a highly-conserved RNA-binding motif that is involved in the post-transcription modification of RNA molecules, such as transfer RNAs (tRNAs) and messenger RNAs (mRNAs). Experimental structures of eukaryotic PUA domains typically reveal a β-barrel structure with a surface curvature where the flipped base of single-stranded RNA (ssRNA) is found within the surface curvature^8–11^. Despite ASCH and PUA domains sharing insignificant sequence similarity, the domains possess a similar fold.

Notably, a structural genomics project recently revealed a novel domain called EVE domain that is present in many prokaryotic proteins^7^. This domain also contains a central β-barrel structure, suggesting that the EVE domain is closely related to the PUA and ASCH domains. However, based on minute structural differences, and on sequence variation compared to PUA and ASCH domains, the authors of that study concluded that EVE proteins might be functionally closer to eukaryotic YT521-B homology (YTH) domains rather than PUA or ASCH domains. Notably, YTH domains also adopt a β-barrel structure and bind RNA molecules with a methylated base^7^, but recent reports revealed their role in mRNA splicing^12^.

In the ethanologenic bacterium *Zymomonas mobilis* ZM4, the gene ZMO0922 encodes a 148 amino acid polypeptide, which has been annotated as a putative ASCH domain-containing protein (*Zm*ASCH) that belongs to family 2^3^. Here, we report the three-dimensional crystal structure of *Zm*ASCH which confirms the fold of a central β-barrel core and reveals a positively-charged cleft with a pocket on the surface. *In vitro* assays showed that *Zm*ASCH bound nucleic acids and possessed cleavage activity specifically towards single-stranded RNAs. The weak nuclease activity of *Zm*ASCH depended on magnesium ions and the protein appeared to prefer the consecutive pyrimidine-adenine dinucleotides for its cleavage activity. Interestingly, *Zm*ASCH cleaved a nucleotide at the 5′-end and its RNA binding or cleavage activity was not affected by the methylation at either the 2′-OH of the ribose ring moiety of or the adenine base near the cleavage site. Based on molecular dynamics simulations, we constructed a model of a solvated RNA:*Zm*ASCH complex and analyzed the interaction between the protein and the bound RNA. This analysis suggested the involvement of key amino acids in RNA interactions and cleavage; their biochemical function *in vitro* was confirmed by site-directed mutagenesis.

## Materials and Methods

### Cloning and protein production

The cloning and purification steps have been described in detail elsewhere^13^. Briefly, the cloned, full-length *Zm*ASCH (ZMO0922) with an N-terminal hexa-His fusion tag was transformed into *E*. *coli* B834(DE3) or *E*. *coli* BL21 Star (DE3) Solu (Novagen, Wisconsin). The expressed protein was purified by sequential chromatographic steps including metal ion affinity, ion-exchange, and size exclusion (SEC). Based on the SEC elution profile, recombinant purified *Zm*ASCH exists as a monomer in solution (Supplementary Fig. 1). For phasing, two leucine residues were successively mutated to methionine residues (L72M, L102M), allowing production of seleno-*L*-methionine (SeMet)-substituted *Zm*ASCH that was prepared in a similar manner to the native protein. Purified protein samples were concentrated to 6 mg/mL after exchanging the buffer to 20 mM Tris-HCl (pH 8.5) and 200 mM NaCl.

### Structure determination

Suitable crystals for diffraction experiments were obtained using two precipitant solutions. The first (precipitation solution 1) contained 40% (w/v) polyethylene glycol (PEG) 400, 0.1 M cacodylic acid (pH 6.5), and 0.2 M lithium sulfate. The second (precipitant solution 2) contained 20% (w/v) PEG 3350, and 0.1 M MES (pH 6.5). For diffraction experiments, crystals were immediately placed in a 100 K nitrogen-gas stream. Indexing, integration, and scaling of the reflections were conducted using the *HKL*2000 suite^14^. The crystals obtained from precipitant solutions 1 and 2 belonged to space groups P3_1_21 and P2_1_, respectively. Using the SeMet-substituted crystal of the double-mutant protein (L72M, L102M), single-wavelength anomalous dispersion data were collected at 0.9793 Å of the Se-peak wavelength on beamline 7A SB1 at the Pohang Accelerator Laboratory (PAL, Republic of Korea), with oscillations of 0.5° and exposures of 0.5 s per frame; a total of 400 images were collected on an ADSC-Q270 CCD detector. Four out of the expected 6 Se sites in the asymmetric unit of the trigonal crystal system were identified at a resolution of 2.75 Å using SOLVE^15^. Electron density modification was performed using PHENIX (Adams 2010)^16^ combined with RESOLVE^17^, resulting in automated modeling of approximately 80% of the residues. Further model building was performed manually using WinCoot^18^, and subsequent refinement was performed using PHENIX^16^. A native data set for the monoclinic crystal system was collected at a wavelength of 1.00 Å on the same beamline with an oscillation of 1° and an exposure of 1 s per frame. The crystal structures of the wild-type protein in the monoclinic system was solved by molecular replacement using the trigonal structure as a search model. Two N-terminal residues could not be located in the trigonal lattice system, whereas all of the residues were located in the monoclinic structure. The two mutated leucine residues, in the α2-helix and the β6-strand, are buried in the hydrophobic core of the globular protein. Mutation of these residues did not result in a noticeable structural change, as indicated by the root-mean-square deviation (RMSD) value of less than 0.25 Å. Analyses of the modeled residues using WinCoot^18^ and MolProbity^19^ demonstrated that all of the residues were in valid regions of the Ramachandran plot (Table 1), with exception of some glycine residues that possessed well-defined electron density yet fall into the disallowed region.

**Table 1:** Data Collection and Structure Refinement Statistics.

Data Collection	High resolution, wild-type	SeMet-Derivative, mutant
Space group	P2_1_	P3_1_21
**Unit cell dimensions**		
a, b, c (Å), α, β, γ (°)	72.91, 52.86, 88.68, 90, 111.56, 90	52.02, 52.02, 206.38, 90, 90, 120
Wavelength (Å)	1.00	0.9793
Resolution (Å)	50–1.7 (1.76–1.7)^a^	30–2.75 (2.80–2.75)^a^
*R*_sym_	10.7 (42.4)	7. 9 (44.8)
*I*/σ(*I*)	17.6 (2.5)	10.2 (2.5)
Completeness (%)	99.1 (98.9)	99.9 (100)
Redundancy	3.4 (3.4)	11.1 (11.2)
Figure of merit SOLVE/RESOLVE		0.37/0.75
**Refinement**		
Resolution (Å)	30.6–1.7 (1.73–1.7)	27.3–2.75 (2.96–2.75)
No. of reflections	68862	16121
*R*_work_/*R*_free_	19.8 (26.1)/23.5 (31.5)	20.0 (24.3)/25.8 (29.9)
**No**. **atoms**		
protein/water	4836/730	2379/51
**RMSD**		
bond lengths (Å)/angles (°)	0.007/1.26	0.005/0.91
**Average B-values (Å** ^**2**^ **)**		
protein/water	24.0/35.0	31.6/25.6
Ramachandran plot (%)		
favored/allowed/outliers	97.4/2.4/0.2	97.9/2.1/0

^a^The numbers in parentheses are the statistics from the highest resolution shell.

### Production of *Zm*ASCH mutants

*Zm*ASCH mutants were generated using a protocol based on the QuickChange II site-directed mutagenesis kit (Agilent Technologies, CA, USA), and mutations were confirmed by DNA sequencing. The mutated codons are underlined and the primer sequences used for site-directed mutagenesis were 5′-GAT GTT AAC ACA ATG TGG AGT CGA TAC-3′ and 5′-GTA TCG ACT CCA CAT TGT GTT AAC ATC-3′ (L72M mutation), 5′-ACG GCT TTT CTC ATG AGA GAC CAT CAG-3′ and 5′-CTG ATG GTC TCT CAT GAG AAA AGC CGT-3′ (L102M mutation), 5′-G CGG ATT TGG ATT TTT GCG ACG CGC CC-3′ and 5′-GG GCG CGT CGC AAA AAT CCA AAT CCG C-3′ (Y47F mutation), 5′-G CGC CCT GTG GAA TCA GTG AT-3′ and 5′-AT CAC TGA TTC CAC AGG GCG C-3′ (K53E mutation), and 5′- G CCA CCC CAG GCT TTG ACG T-3′ and 5′-A CGT CAA AGC CTG GGG TGG C-3′ (S128A mutation). The mutant proteins were purified using the same procedure as that used for the wild-type protein.

### Electrophoretic mobility shift assay (EMSA)

The sequences of the different nucleic acids used in this study are tabulated in Table 2. The synthesized RNA strands were purchased from ST Pharm (Republic of Korea). The 5′-ends of the RNA and DNA molecules were labeled with [γ-^32^P]-ATP using T4 polynucleotide kinase (Roche, Germany). To remove unincorporated [γ-^32^P]-ATP, the mixture was desalted using an RNase-free Sephadex G-25 column (GE Healthcare, Sweden). The labeled probes were then incubated with *Zm*ASCH for 30 min at 310 K in a buffer solution consisting of 20 mM Tris–HCl (pH 7.5), 10 mM magnesium acetate, 300 mM potassium chloride, 100 ng/μL bovine serum albumin, and 100 ng/μL heparin. The mixtures were loaded onto 15 or 20% (w/v) non-denaturing polyacrylamide gel (40:1). Electrophoresis was conducted at 70 V for 60 min at 298 K in Tris-borate-EDTA buffer (89 mM Tris, 89 mM boric acid, and 2 mM EDTA). The results were visualized by using a Fuji phosphorimager.

**Table 2:** Oligonucleotides used in this study.

	Sequences
ssRNA(30mer)	5′-AUCAGCUCGUCACAACAUUACUUCAUCAAC-3′ (Figs 3a,b and 4d)
ssDNA(30mer)	5′-ATCAGCTCGTCACAACATTACTTCATCAAC-3′ (Figs 3a and 4d)
probe 1, ssRNA(17mer)	5′-CCCGAC^∨^AAC^∨^AGGCCCCC-3′ (Fig. 3c)
probe 2, ssRNA(17mer)	5′-CCCC^∨^AU^∨^AAU^∨^AGGCCCCC-3′ (Fig. 3c)
probe 3, ssRNA(17mer)	5′-CCCGAC^∨^AGCCGGCCCCC-3′ (Fig. 3d)
probe 4, ssRNA(17mer)	5′-CCCGAC^∨^A(m)GCCGGCCCCC-3′ (2′-Ome, Fig. 3d)

The methylated nucleotide is indicated with “m” in the parenthesis, while the theoretical cleavage sites of ssRNA (17mer)s are indicated with “^∨^”.

### Nuclease activity assay

Radioactively labeled DNA or RNA probes were mixed with *Zm*ASCH for 30 min at 310 K, in either the presence or absence of divalent metal ions, in a buffer solution consisting of 20 mM Tris-HCl (pH 8.0), 100 mM potassium chloride, and 100 ng/μL bovine serum albumin. The mixtures were analyzed by EMSA, as described above.

### Molecular dynamics simulation

All molecular dynamics simulations (*Zm*ASCH in water, *Zm*ASCH:RNA in water) were carried out with Gromacs 4.6.5 using the AMBER03 protein, AMBER94 nucleic acid, and the TIP3P water model^20^. A hepta-nucleotide RNA molecule (AGGACAU) obtained from PDB ID 4u8t^21^ was manually placed in the binding site to obtain a starting conformation for the simulations. Sodium and chloride ions were added by replacing solvent molecules at sites of high electrostatic potential to ensure a charge-neutral cell and at a concentration of 100 mM. Following an energy minimization step, a position-restrained dynamics simulation of 20 ps with a simulation time step of was performed to gradually equilibrate the solvated protein:RNA complexes at 300 K and 1 bar. Periodic boundary conditions were applied in all three dimensions. Long-range interactions were modeled using the particle mesh Ewald method^22^ and a grid spacing of 1.2 Å; the cutoff for computation of short-range electrostatic interactions was 10 Å, and 14 Å for van der Waals interactions. The temperature was controlled with the V-rescale thermostat^23^ and the pressure with the Parinello-Rahman barostat^24^. All bonds were constrained using the LINCS algorithm^25^. The final MD simulation was performed for 20 ns with a time step of 0.001 ps. Simulations were performed on a custom-built server with an Intel Xeon E5-1650 Six Core (3.5 GHz) and 32 GB RAM. Analyses were performed with Gromacs tools and automated plots generated with Grace (http://plasma-gate.weizmann.ac.il/Grace/).

## Results and Discussion

### The fold of *Zm*ASCH is conserved among ASCH proteins

**Figure 1:**
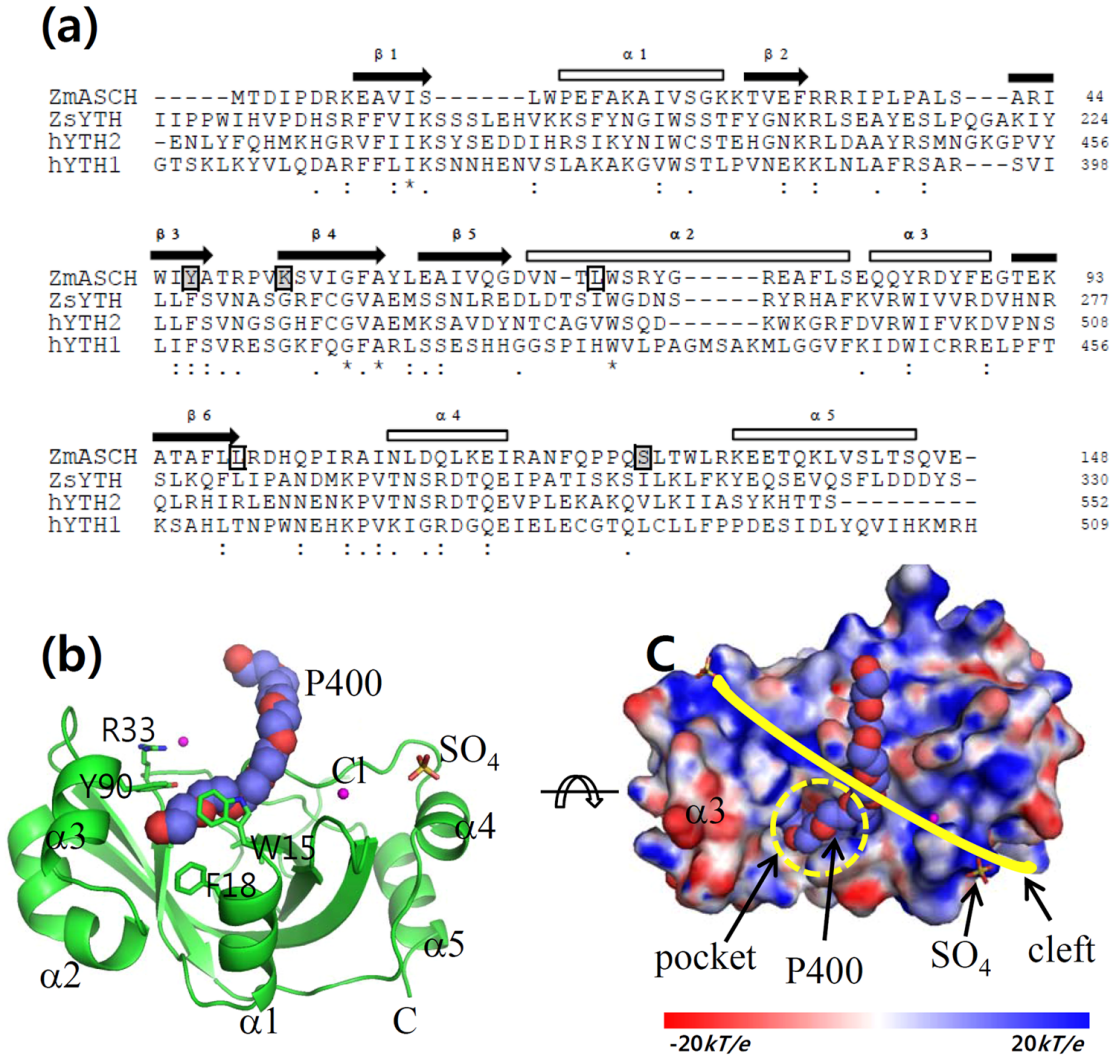
Crystal structure of *Zm*ASCH. (**a**) Structure-based sequence alignment of *Zm*ASCH with other related proteins. Sequence alignment was performed using the Clustal Omega from the European Bioinformatics Institute. Identical residues are marked with an asterisk “*”, while strongly and weakly similar residues are marked with “:” and by “.”, respectively. The α-helices (cylinders) and β-strands (arrows) in the *Zm*ASCH structure are numbered in order of their appearance. The Leu residues that were mutated to solve the phasing problem are indicated with boxes. Residues that were mutated for *in vitro* assays are enclosed in black boxes over a gray background. Abbreviations: *Zs*YTH, YTH from *Z*. *rouxii*; hYTH1, human YTH1; hYTH2, human YTH2; *Zm*ASCH, ASCH from *Z*. *mobilis* ZM4. (**b**) A ribbon diagram of *Zm*ASCH with non-protein molecules observed in the trigonal crystal system. The putative chloride ions (Cl) are represented by pink balls, and the bound polyethylene glycol 400 (P400) is shown as a space-filling model. The observed sulfate ion (SO_4_), and some residues that form the pocket and the surface cleft, are drawn as stick models. (**c**) Surface-potential map of the *Zm*ASCH molecule in Fig. 1b. The surface electrostatic potential was calculated using the APBS server (http://www.poissonboltzmann.org/) and is visualized as a color ramp from blue (positive) to red (negative). This figure was rotated with respect to Fig. 1b, to clearly display the surface-potential in the pocket and on the nucleic acid-binding cleft. The surface pocket is marked with a yellow-dotted circle (pocket), and the positively-charged cleft on the surface is indicated with a red-dotted line (cleft). The bound P400 is depicted as a space-filling model and the sulfate ion (SO_4_) is depicted as stick models. Figures 1(a), 2, 4(a),(b),(c) were prepared by the PyMol molecular graphics program (Schrödinger, LLC).

The structure of *Zm*ASCH protein comprises five α-helices and a six-stranded β-sheet (Fig. 1a,b), the latter forming the central β-barrel. This β-barrel is flanked by two pairs of antiparallel α-helices (α2/α3 and α4/α5) as well as the single helix α1. A deep pocket exhibiting a positively-charged protein surface is formed between helices α1, α3 and the central β-barrel (Fig. 1c). There are two and four protein molecules in the asymmetric unit of the trigonal and monoclinic crystal forms, respectively. The conformations of individual monomers are in excellent agreement, and all monomers superimpose with RMSD values of less than 0.3 Å, with the largest deviations in the loops between strands β2 and β3 and helices α4 and α5. In the trigonal crystal form, the positively charged pocket harbors a PEG400 molecule as well as chloride and sulfate ions (Fig. 1b).

The overall structure of *Zm*ASCH protein superimposes well on other ASCH family proteins (Supplementary Fig. 2), with low RMSD values. The GxKxxxxR motif, conserved among ASCH proteins, as well as the notable surface pocket occupy spatially equivalent positions. Therefore, the fold and structural features known from other ASCH families are well conserved in this family 2 protein. Sequence variation of the aligned ASCH proteins is found on the outside helices on the compared structures (Supplementary Fig. 2).

### *Zm*ASCH has many non-ASCH structural homologues

**Figure 2:**
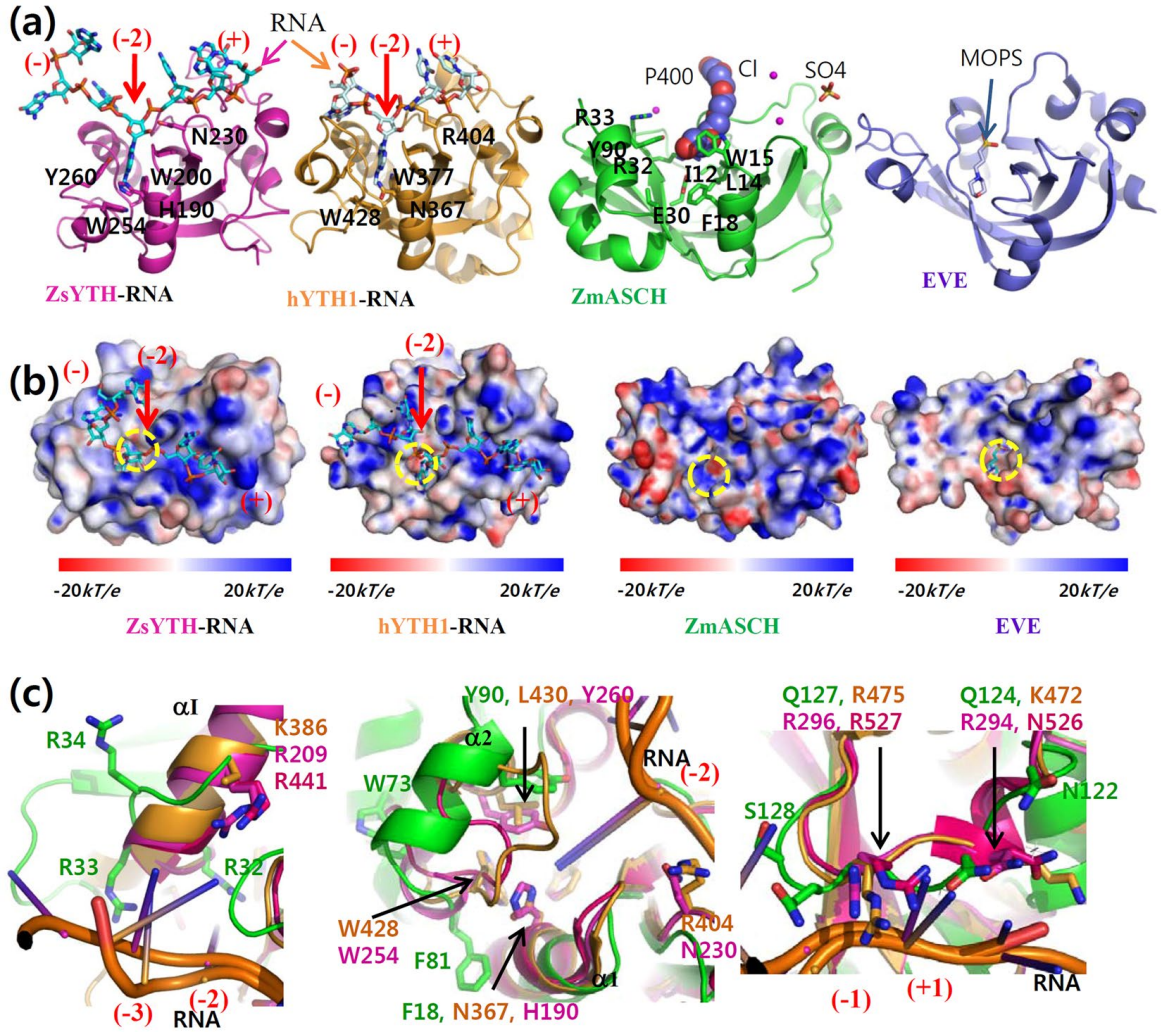
Comparison of *Zm*ASCH with related structures. (**a**) Four structurally related proteins. The RNA molecule with a modified adenine base in the protein pocket, and the residues around the pocket, are represented with stick models, together with the protein molecules (*Zs*YTH, magenta; hYTH1, gold). The positions of the RNA nucleotides are labeled with negative numbers to 5′-end, and positive numbers to 3′-end relatively to the cleavage site. The structurally related prokaryotic proteins are displayed with the bound non-protein molecules (*Zm*ASCH with P400 as a space-filling model, green; EVE domain protein with 3-(*N*-morpholino) propanesulfonic acid (MOPS) shown as a stick model, marine). (**b**) Surface profiles. The surface potential of the compared proteins was mapped with coloring schemes for the electrostatic potentials. The bound RNA molecules in *Zs*YTH and hYTH1, each bearing a methyl group, are drawn as stick-models. The protein pockets binding the methylated-adenine base are marked with yellow-dotted circles. The pockets of *Zm*ASCH and the EVE domain protein are also indicated with yellow-dotted circles. (**c**) Close-up view of three regions. The regions interacting with −4 and −3 (left), −2 (middle), −1 and +1 (right) sites of the RNA molecules were magnified after structural superimposition. Some key residues are depicted as stick models in different colors: *Zm*ASCH (green), hYTH1 (orange), hYTH2 (red), and *Zs*YTH (magenta).

A search for structurally similar proteins using the DALI server (http://ekhidna.biocenter.helsinki.fi/dali_server/) showed that the three-dimensional structure of *Zm*ASCH is closely related to that observed with several proteins. As expected, these proteins include a prokaryotic EVE protein^7^ (PDB ID 2eve; Z-score 11.2; RMSD 2.4 Å); human ASC-1 (PDB ID 2e5o; Z-score 10.2; RMSD 3.2 Å); thymocyte nuclear protein 1^26^ (PDB ID 3eop; Z-score 9.2; RMSD 3.0 Å); and several proteins bearing YTH domains: the YTH domain from *Zygosaccharomyces rouxii* MRB1 protein^21^ (PDB ID 4u8t; Z-score 9.7; RMSD 2.5 Å; hereafter called *Zs*YTH), human YTHDC1 protein^27^ (PDB ID 4r3i; Z-score 9.7; RMSD 2.7 Å; hereafter called hYTH1), and human YTHDF2 protein^28^ (PDB ID 4rdo; Z-score 9.3; RMSD 2.7 Å; hereafter called hYTH2) (Fig. 2a,b,c). Among these different proteins, the electric potential of the protein surface is also well conserved along the cleft (Fig. 2b).

There are, however, several differences noticeable upon superimposition of *Zm*ASCH and the structural homologues. First, compared to *Zm*ASCH, *Zs*YTH and hYTH1 have ~15 additional residues at the N-terminus (Fig. 1a). These residues are far from the RNA-binding site and the methylated-adenine binding pocket in the YTH structures^21,27,28^, suggesting their limited role, if any, in the molecular function of RNA recognition. A second difference is found in the loop between strands β2 and β3 (Arg33-Leu40) in *Zm*ASCH; that loop is elongated in *Zs*YTH and hYTH and it contains conserved RNA-interacting residues (Arg209 in *Zs*YTH; Lys386 or Arg441 in the hYTH proteins) (Fig. 2c, left)^21,27,28^. A third structural difference is found in helices α2 and α3 which harbor several aromatic residues (Trp73, Tyr76, Phe81, Tyr87, Tyr90, and Phe91) (Fig. 1). This region is shorter and flexible in *Zs*YTH (Leu249-Arg259), but it provides important aromatic residues (Trp254 and Tyr260) that recognize the methylated-adenine base^21^. hYTH1 forms a short α-helix in the corresponding structural region^25^, but lacks aromatic or positively charged residues. A fourth difference is found in the loop (Ala121-Lys134) between helices α4 and α5 in *Zm*ASCH, which is structurally equivalent to the elongated regions in *Zs*YTH (Leu283-Pro302) and hYTH1 (Thr462-Leu482) that provide two key residues (Asn294 and Arg296 in *Zs*YTH; Lys472 and Arg475 in hYTH1; Asn524 and Arg527 in hYTH2) that interact with the RNA phosphate backbone and a hydroxyl group on the ribose ring moiety^21,27^. Although these residues are not conserved in *Zm*ASCH at the same site, potential polar residues (Asn122, Gln124, Gln127, and Ser128), which might possibly interact with RNA molecules, are found nearby (Fig. 2c, right).

In summary, comparison of the newly determined *Zm*ASCH structure with related protein structures shows that ASCH, EVE, PUA, and YTH proteins share a central β-barrel with some differences in the flanking α-helices (Fig. 2). Notably, all of these proteins also form a pocket, either closed or partially open, between the central β-barrel and the surrounding α-helices.

### *Zm*ASCH is a nuclease with specificity for single-stranded RNA molecules

Owing to a characteristic surface curvature as a result of the underlying β-barrel structure, eukaryotic PUA domains posses the ability to bind ssRNA^8–11^. Additionally, a short RNA fragment with a methylated adenine base is found in the positively charged cleft on the protein surface of YTH proteins. The methylated base is trapped in the hydrophobic pocket on the surface cleft (Fig. 2a,b), where aromatic residues of proteins interact with a methyl moiety on the base at one side or both sides^21,28^. A long cleft (indicated by the yellow line in Fig. 1c) is formed at one side of the *Zm*ASCH molecule. Similar to the observation with YTH proteins, the electrostatic surface potential map of *Zm*ASCH shows that this cleft has an almost continuous positive surface potential (Fig. 2b). Importantly, this cleft encompasses the pocket.

**Figure 3:**
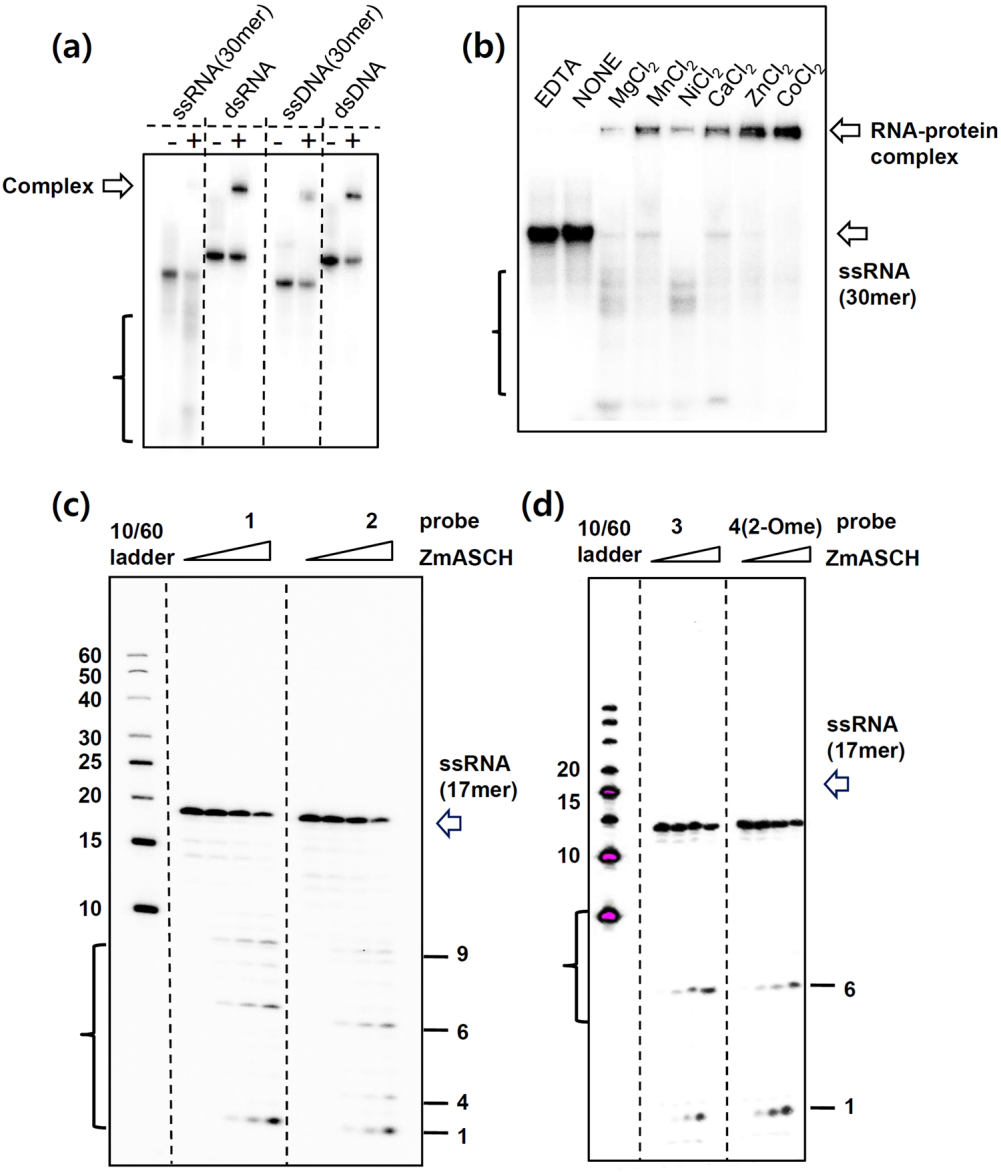
*Zm*ASCH exhibits a ribonuclease activity. (**a**) EMSA with four types of nucleic acids. Each of the indicated nucleic acids (3 nM) was incubated with wild-type *Zm*ASCH (300 nM). EMSA was performed on a 15% (w/v) non-denaturating polyacrylamide gel. (**b**) ^32^P-labelled 30-mer RNA (3 nM) was incubated with *Zm*ASCH (300 nM) for 30 min at 310 K in the absence or presence of 10 mM MgCl_2_, MnCl_2_, NiCl_2_, CaCl_2_, ZnCl_2_ or EDTA. EMSA was performed on a 15% (w/v) non-denaturating polyacrylamide gel. (**c**,**d**) The indicated RNA probes (3 nM) were incubated with wild-type *Zm*ASCH protein (0, 150, 300, 500 nM) for 30 min at 310 K in the presence of 10 mM MgCl_2_. EMSA was performed on a 20% (w/v) non-denaturating polyacrylamide gel. The brackets (**a**–**d**) indicate the degradation products.

Based on this conserved structural feature in both YTHs and *Zm*ASCH, we tested whether *Zm*ASCH possessed nucleic acid-binding properties using an *in vitro* electrophoretic mobility shift assay (EMSA). The assay results clearly demonstrated that *Zm*ASCH binds nucleic acids (Fig. 3a), but we also observed unexpected degradation of single-stranded (ss) RNA in the presence of magnesium ions. This weak ribonucleolytic activity was observed in the presence of either Mn^2+^ or Ni^2+^, but not with Zn^2+^ or Co^2+^ (Fig. 3b). Since no nuclease activity was detected towards ssDNA, double-stranded (ds) DNA, or dsRNA (Fig. 3a), we concluded that *Zm*ASCH is a ribonuclease with specificity for ssRNA.

Careful analysis of the endo-ribonucleolytic degradation pattern of ssRNA substrates with random sequence indicates that *Zm*ASCH preferably cleaves the phosphodiester bond between cytosine and adenosine or uridine and adenosine nucleotide. Hence, we synthesized three ssRNAs of 17 nucleotides with one to three theoretical cleavage sites, respectively (Fig. 3c,d, Table 2). Besides the expected endo-ribonucleolytic degradation products, unexpected cleavage products of single nucleotides from the 5′-ends were also observed with all the ssRNAs used (Fig. 3c,d). The exo-nucleolytic activity was observed with two different 5′-end nucleotides of adenine and cytidine (Supplementary Fig. 3). Hence it appears that the enzyme does not discern at least the pyrimidine and purine base at the 5′-end. The RNA-degrading activity of *Zm*ASCH was also detected with RNA substrates bearing a methylated nucleotide at the cleavage site (methylation either at the nitrogen atom or 2′-OH; Supplementary Fig. 4 and right panel in Fig. 3d), indicating that methylation at the +1 site does not affect the endo-nucleolytic activity.

In summary, we conclude that *Zm*ASCH is a ribonuclease with endo- as well as exo-nucleolytic activity. Whereas the endo-nuclease activity of *Zm*ASCH was sequence-specific, the exo-nuclease activity was 5′-end-specific without discrimination of purine or pyrimidine nucleotide.

### The ribonucleolytic site of *Zm*ASCH is located in the basic cleft

As the ssRNA-cleavage activity of *Zm*ASCH was dependent on magnesium ions, but inhibited by other transition metal ions, such as zinc or cobalt, co-crystallization of the wild-type protein with either ZnCl_2_ or CoCl_2_ was attempted. However, we could not locate any bound transition metal ions in either crystal form, suggesting that high affinity binding of the metal ion to the protein molecule is probably accomplished in the presence of a nucleic acid. We further tried to co-crystallize the wild-type protein with various ssRNA and ssDNA samples but so far failed to obtain crystals of such complexes.

**Figure 4:**
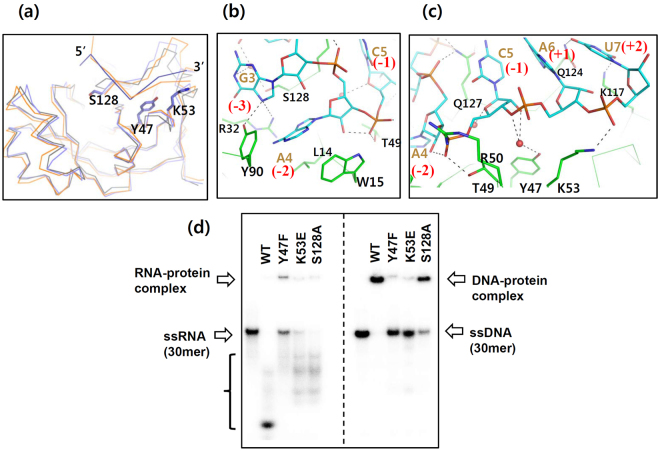
Ribonucleolytic site of *Zm*ASCH. (**a**) Simulated *Zm*ASCH structure in complex with an ssRNA AGGACAU. The *Zm*ASCH *apo*-structure and two representative simulated complex structures were superimposed. Three protein structures were displayed with coils of different colors. The bound ssRNA is depicted as blue lines; 5′- and 3′-ends are labeled. Some key residues that interact with ssRNA, which were mutated, are depicted as stick models. (**b**,**c**) Interaction of *Zm*ASCH with an ssRNA at two sites. The bound ssRNA (cyan-colored carbon) and the protein residues (green-colored carbon) were drawn as thick stick models. Water molecules are displayed as red spheres and polar interactions among atoms are indicated with black-dotted lines. (**d**) ^32^P-labelled RNA or DNA probes (0.3 μM) were incubated with wild-type and mutant proteins of *Zm*ASCH (3 μM) for 30 min at 310 K in the presence of 10 mM MgCl_2_. The bracket on the left panel indicates the degradation products.

To gain an insight into the interaction between *Zm*ASCH and RNA, we performed a molecular dynamics simulations study of the solvated protein in the presence of a hepta-nucleotide RNA molecule (AGGACAU) as a possible substrate (Supplementary Fig. 5). For the simulation, the RNA molecule was placed in the *Zm*ASCH cleft, and the resulting complex protein was solvated. The initial position of the RNA molecule was guided by superposition of *Zm*ASCH with a complex of *Zs*YTH with a surface-bound RNA fragment that includes a methylated-adenine base in the surface pocket^21^. In the simulated complex structure, the RNA molecule binds to the encompassing positively charged surface cleft of *Zm*ASCH (Fig. 4a). The adenine base (A4) of the bound RNA is situated in the hydrophobic pocket formed by non-polar residues on the surface (Leu14, Trp15, and Tyr90) (Fig. 4b). Thr49, Arg50, Lys53, and Gln127 directly interact with atoms of the phosphate backbone, while Tyr47 forms an interaction with the phosphate moiety between the −1 and +1 nucleotides through the intervening water molecules (Fig. 4c). The bases of the bound ssRNA interact with the side-chain atoms of Arg32, Tyr90, Lys117, Gln124, and Ser128. Gln127 forms an elaborate interaction network with two phosphate moieties and the hydroxyl group of a ribose moiety (Fig. 4c). Since results from the nuclease activity assays suggested that endo-nucleolytic cleavage occurs at the phosphodiester bond between the pyrimidine and the adenine nucleotides, cleavage of the RNA fragment in the simulation should occur at the phosphodiester bond between the −1 cytidine (C5) and the +1 adenine (A6) nucleotides. This assumption is in agreement with the binding pose observed in the simulation (Fig. 4c).

Based on these simulation results (Fig. 4a) and extensive analysis of amino acid sequences of related proteins (Supplementary Fig. 6), we introduced a charge-reversal mutation at the site of Lys53 (K53E), within the positively charged surface cleft. This mutant exhibited reduced binding to ssDNA and ssRNA molecules (Fig. 4d). Another mutation at Ser128 (S128A) also decreased the binding of *Zm*ASCH to nucleic acids, although to a lesser degree (Fig. 4d). However, removal of the side-chain hydroxyl group in Tyr47 (Y47F) substantially decreased the DNA- and RNA-binding activity of the protein. In addition, this Y47F mutant also possessed decreased RNA-hydrolytic activity (Fig. 4d). Importantly, this tyrosine residue is conserved in the EVE proteins^7^. Mutations of the two independent residues at the positions of 47 and 128 did not make a noticeable structural change (Supplementary Fig. 7 and Table S2).

Since the wild-type *Zm*ASCH protein did not degrade the bound ssDNA (Fig. 4d), it seems likely that the presence of an oxygen atom at the 2′-site of the sugar ring of the bound substrate, probably at the −1 site, is required for the nucleolytic activity of the protein.

Frequently, catalytic metal ions for nucleolytic reactions are coordinated by acidic residue side chains of protein molecules^29,30^. Because the RNA-binding surface harbouring the proposed nucleolytic site of *Zm*ASCH has no significant negatively charged surface (Fig. 1b) and no conserved acidic amino acid residues in this cleft (Supplementary Fig. 6b), it remains unclear how the required divalent metal ion is bound by *Zm*ASCH. We hypothesize that protein main-chain carbonyl oxygen atoms as well as hydroxyl groups of the bound RNA molecule provide coordination for the catalytically required magnesium ion and a metal-bound water molecule acts as a nucleophile, reminiscent of the situation found in other hydrolases, such as for example trehalose-6-phosphate phosphatase from fungi^31^. Clearly, future studies need to address the experimental structure of *Zm*ASCH in complex with RNA and a metal ion such as to clarify the precise substrate binding pose and cleavage mechanism of this *Zm*ASCH ribonuclease.

### Possible cellular roles for ASCH

In this study, we have shown that *Zm*ASCH has conserved structural features among ASCH, EVE, PUA, and YTH proteins and binds nucleic acids using the positively charged surface cleft. The protein possesses ribonucleolytic activity towards ssRNA with a preference for the phosphodiester bond formed between the pyrimidine and adenine nucleotides. It also removes the terminal nucleotide at the 5′-end. The enzyme did not discriminate the methylation state at the 2′-OH of the +1 adenine nucleotide at the cleavage site. However, it remains unclear whether the methylation at the 2′-OH of the −1 site affects the endo-nucleolytic activity. Since only two methylated probes have been investigated, it cannot totally be excluded that *Zm*ASCH can recognize the methylation of a specific sequence in the substrate, for example, at the −1 site. A more extensive future study of methylated substrate processing by the prokaryotic YTH homolog *Zm*ASCH will clarify this aspect and its cellular implications.

A cellular role for the human protein, hYTH1, has recently been reported as a protein involved in pre-mRNA splicing together with factors SRSF3 and SRSF10^12^. Given that mRNA splicing in prokaryotes has not been reported, the cellular role of prokaryotic structural homologues of hYTH1, such as the ASCH and EVE proteins, might need careful reconsideration. The unexpected ribonucleolytic activities of *Zm*ASCH against ssRNAs suggest that the protein may participate in removal of cellular RNAs. In this context, it is particularly noteworthy that removal of 5′-phosphate in bacterial mRNAs lacking the Shine-Dalgarno sequence decreased the *in vivo* abundance of full-length mRNA and hindered *in vivo* translation^32^, suggesting that many unidentified ribonucleolytic enzymes might be involved in controlling the cellular RNA amount. The search for the real cellular substrate of *Zm*ASCH, for example, by transcriptomic analysis will give a clue for its exact physiological roles. ASCH domains have high sequence homology with ribosomal proteins and the Cro/Cl family of transcriptional regulators (Supplementary Fig. 6a) and most of the strictly conserved residues including Tyr47, the unique residue that is structurally conserved in most of EVE proteins^7^, are found around the cleft and the surface pocket in the structure (Supplementary Fig. 6b), suggesting that *Zm*ASCH is involved in the transcription and translation. Notably, ASCH was initially reported as a domain of ASC-1 that is a transcriptional regulator of nuclear receptors and a component of a putative RNA-interacting protein complex^1,2^.

### Data deposition

Atomic coordinates were deposited in the Protein Data Bank, http://www.rcsb.org (accession numbers 5GUQ, 5GUS, 5Y6B, and 5Y6C).

## Electronic supplementary material

Supplementary Fig. 1

## References

[1] Kim HJ (1999). Activating signal cointegrator 1, a novel transcription coactivator of nuclear receptors, and its cytosolic localization under conditions of serum deprivation. Mol. Cell Biol..

[2] Jung D-J (2002). Novel Transcription Coactivator Complex Containing Activating Signal Cointegrator 1. Mol. Cell Biol..

[3] Iyer LM, Burroughs AM, Aravind L (2006). The ASCH superfamily: novel domains with a fold related to the PUA domain and a potential role in RNA metabolism. Bioinformatics.

[4] Kim SH (1998). Shining a light on structural genomics. Nat. Struct. Mol. Biol..

[5] Sali A (1998). 100,000 protein structures for the biologist. Nat. Struct. Mol. Biol..

[6] Teichmann SA, Chothia C, Gerstein M (1999). Advances in structural geomics. Curr. Opin. Struct. Biol..

[7] Bertonati C (2009). Structural genomics reveals EVE as a new ASCH/PUA-related domain. Proteins.

[8] Ishitani R (2003). Alternative tertiary structure of tRNA for recognition by a posttranscriptional modification enzyme. Cell.

[9] Pan H (2003). Structure of tRNA pseudouridine synthase TruB and its RNA complex: RNA recognition through a combination of rigid docking and induced fit. Proc. Natl. Acad. Sci. USA.

[10] Li L, Ye K (2006). Crystal structure of an H/ACA box ribonucleoprotein particle. Nature.

[11] Liang B (2009). Structure of a functional ribonucleoprotein pseudouridine synthase bound to a substrate RNA. Nat. Struct. Mol. Biol..

[12] Xiao W (2016). Nuclear m(6)A Reader YTHDC1 Regulates mRNA Splicing. Mol. Cell.

[13] Park SY, Park JH, Kim JS (2011). Cloning, expression, purification, crystallization, and preliminary X-ray diffraction analysis of an ASCH domain-containing protein from. Zymomonas mobilis ZM4. Acta. Crystallographica F.

[14] Otwinowski Z, Minor W (1997). Procession of x-ray diffraction data collected in oscillation mode. Methods Enzymol..

[15] Terwilliger TC (1999). Automated MAD and MIR structure solution. Acta Crystallogr. D Biol. Crystallogr..

[16] Adams PD (2010). PHENIX: a comprehensive Python-based system for macromolecular structure solution. Acta Crystallographica.

[17] Terwilliger TC, Berendzen J (2000). Maximum-likelihood density modification. Acta Crystallogr. D Biol. Crystallogr..

[18] Emsley P, Cowtan K (2004). Coot: model-building tools for molecular graphics. Acta Crystallographica.

[19] Davis IW (2007). MolProbity: all-atom contacts and structure validation for proteins and nucleic acids. Nucleic Acids Res..

[20] Duan Y (2003). A point-charge force field for molecular mechanics simulations of proteins based on condensed-phase quantum mechanical calculations. J. Comput. Chem..

[21] Luo S, Tong L (2014). Molecular basis for the recognition of methylated adenines in RNA by the eukaryotic YTH domain. Proc. Natl. Acad. Sci. USA.

[22] Darden T, York D, Pedersen L (1993). Particle mesh Ewald: An N⋅log(N) method for Ewald sums in large systems. J. Chem. Phys..

[23] Bussi G, Donadio D, Parrinello M (2007). Canonical sampling through velocity rescaling. J. Chem. Phys..

[24] Parrinello M, Rahman A (1981). Polymorphic transitions in single crystals: A new molecular dynamics method. J. Chem. Phys..

[25] Hess B, Bekker H, Berendsen HJC, Fraaije JGEM (1997). LINCS: a linear constraint solver for molecular simulations. J. Comp. Chem..

[26] Yu F (2009). Determining the DUF55-domain structure of human thymocyte nuclear protein 1 from crystals partially twinned by tetartohedry. Acta Crystallogr. Sect.D.

[27] Xu C (2014). Structural basis for selective binding of m6A RNA by the YTHDC1 YTH domain. Nat. Chem. Biol..

[28] Li F, Zhao D, Wu J, Shi Y (2014). Structure of the YTH domain of human YTHDF2 in complex with an m(6)A mononucleotide reveals an aromatic cage for m(6)A recognition. Cell Res..

[29] Beloglazova N (2011). Structure and activity of the Cas3 HD nuclease MJ0384, an effector enzyme of the CRISPR interference. EMBO J..

[30] Gong B (2014). Molecular insights into DNA interference by CRISPR-associated nuclease-helicase Cas3. Proc. Natl. Acad. Sci. USA.

[31] Miao Y (2016). Structures of trehalose-6-phosphate phosphatase from pathogenic fungi reveal the mechanisms of substrate recognition and catalysis. Proc. Natl. Acad. Sci. USA.

[32] Giliberti J, O’Donnell S, Etten WJ, Janssen GR (2012). A 5′-terminal phosphate is required for stable ternary complex formation and translation of leaderless mRNA in Escherichia coli. RNA.

