# Supplementary material for: Crystal structure of an ASCH protein from Zymomonas mobilis and its ribonuclease activity specific for single-stranded RNA
Source: Sci Rep. 2017 Sep 26;7:12303. doi: 10.1038/s41598-017-12186-w (PMC5615036; doi:10.1038/s41598-017-12186-w)
Supplement: Supplementary file 1 — Supplementary Fig. 1 [file 41598_2017_12186_MOESM1_ESM.pdf]

Supplementary Information

Crystal structure of an ASCH protein from *Zymomonas mobilis* and its ribonuclease activity specific for single-stranded RNA

Bo-Na Kim, Minsang Shin, Sung Chul Ha, Suk-Youl Park, Pil-Won Seo, Andreas Hofmann, and Jeong-Sun Kim

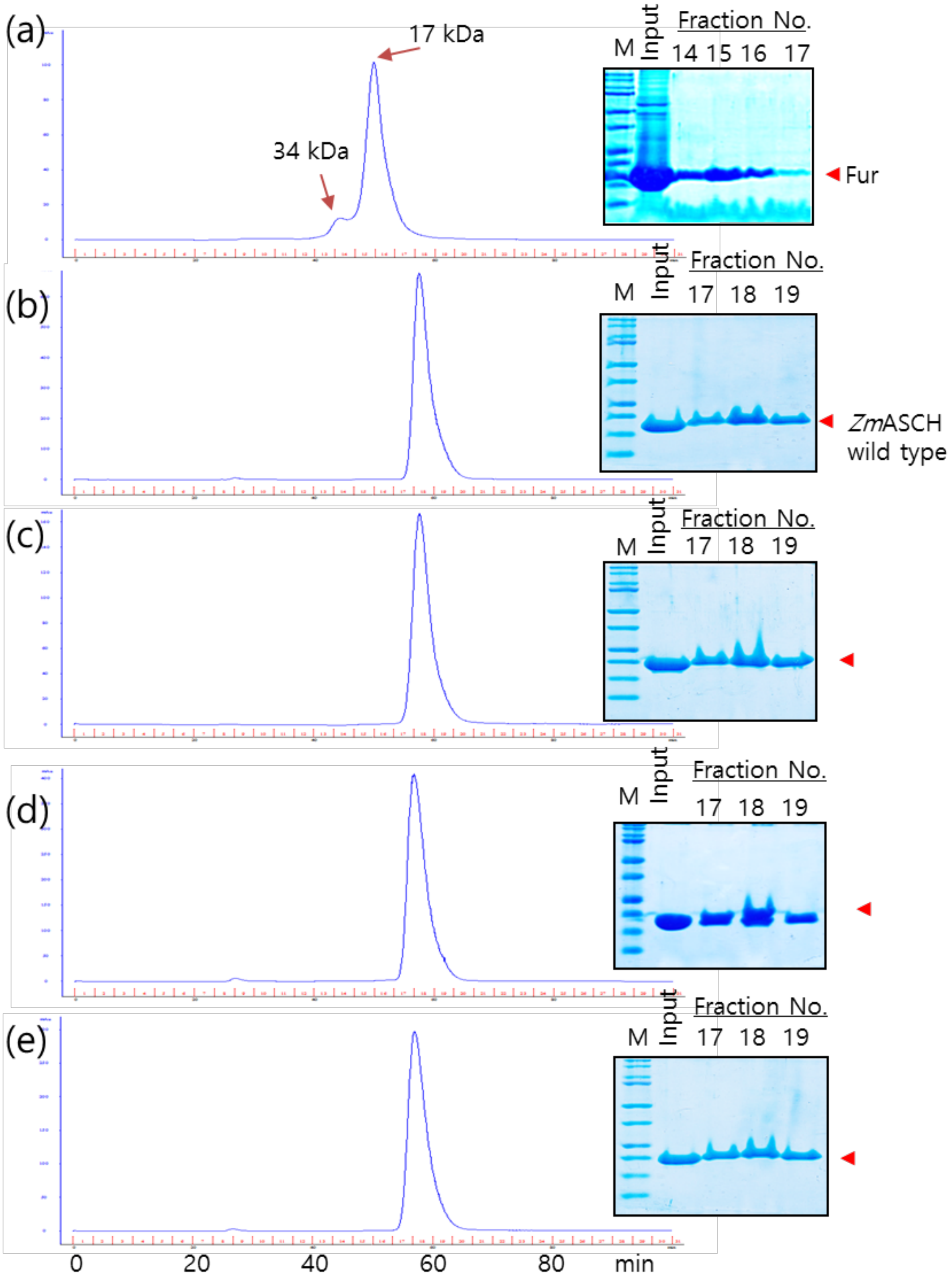

Supplementary Fig. 1. Size exclusion chromatography (SEC). The SEC was performed with an AKTA Prime chromatography system by using a Superdex 200 SEC (16/600; GE Healthcare) in a buffer consisting of 20 mM Tris·HCl at pH 7.5 and 100 mM NaCl at a flow rate of 1.5 mL/min. (a) apo-ferric uptake regulator (Fur, 17 KDa), (b) *Zm*ASCH wild-type, (c) *Zm*ASCH Y47F, (d) *Zm*ASCH K53E, and (e) *Zm*ASCH S128A. The chromatograms were obtained by monitoring the absorbance at 280 nm. Tricine-SDS/PAGE gel with molecular mass standard marker (Bio-Rad) ranging from 10 to 170 kDa and the eluted fractions.

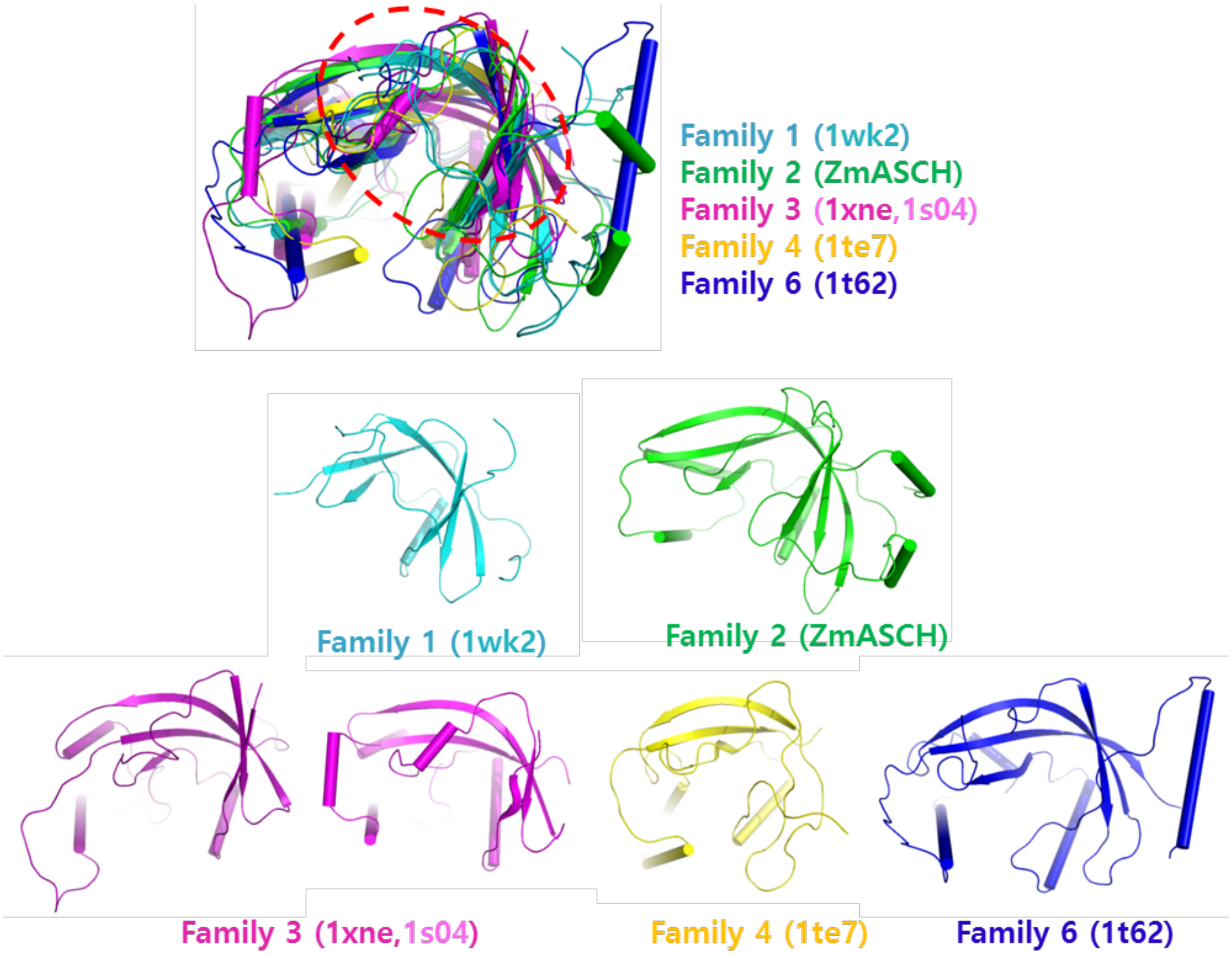

Supplementary Fig. 2. Comparison of ASCH domain structures. The α-helices and β-strands are represented as cylinders and arrows, respectively. Each structure is differentiated by alternating colors. The central β-barrel was indicated with a red-dotted circle in the top panel.

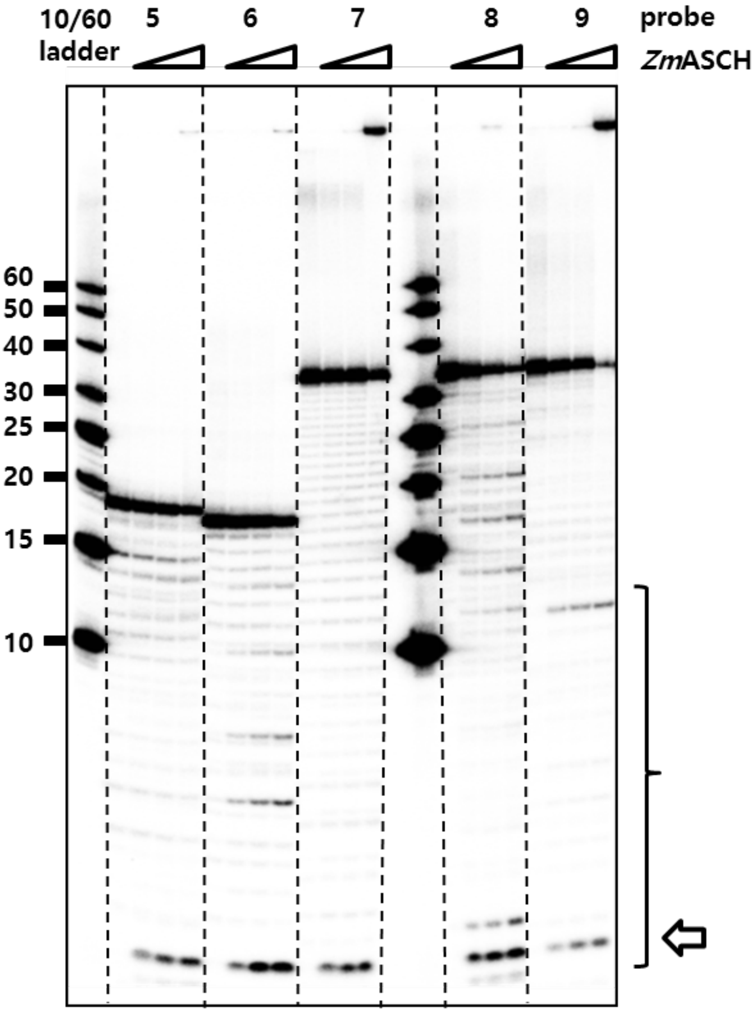

Supplementary Fig. 3. EMSA with various RNA sequences. Each RNA probe (3 nM) was incubated with wild-type *Zm*ASCH protein (0, 150, 300, 500 nM) for 30 min at 310 K in the presence of 10 mM $MgCl_2$ and analyzed with 20 % (w/v) non-denaturating polyacrylamide gel. The degradation products are indicated with a bracket and the position of a single nucleotide is with an arrow.

Supplementary Table S1. Oligonucleotide substrates used in this study

Substrate	Sequence
probe 5, RNA (16-mer)	5'-AUGAGCUCGU CACAAG-3'
probe 6, RNA (16-mer)	5'-AGAUUACAAC AACAAC-3'
probe 7, RNA (30-mer)	5'-AAAAAAAAAA AAAAAAAAAA AAAAAAAAAA-3'
probe 8, RNA (30-mer)	5'-AUCAGCUCGU CACAACAUUA CUUCAUCAAC-3'
probe 9, RNA (30-mer)	5'-GUUGUUGUUG UAAUCUUGUG ACGAGCUCAU-3'

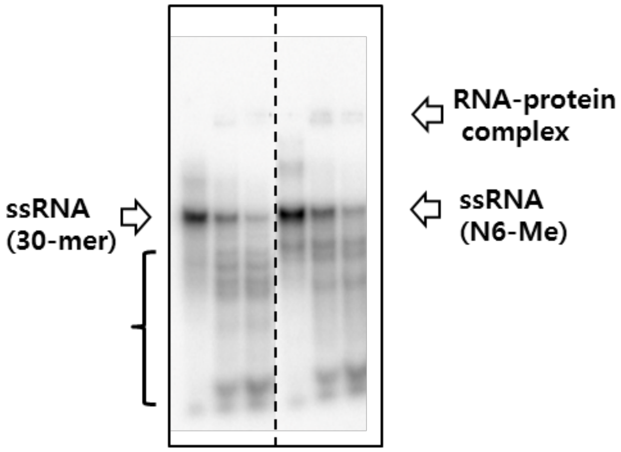

Supplementary Fig. 4. *Zm*ASCH cleaves the probe with a methyl group at the N6-atom of the adenine nucleotide. The indicated RNA probe (3 nM) was incubated with wild-type *Zm*ASCH protein (0, 150, 300 nM) for 30 min at 310 K in the presence of 10 mM $MgCl_2$ and analyzed with 15% (w/v) non-denaturing polyacrylamide gel. The bracket indicates the degradation products. Sequence for the probe is 5′-AUGAGCUCGUCACAGGA(m)CUACAACAACAAC-3′.

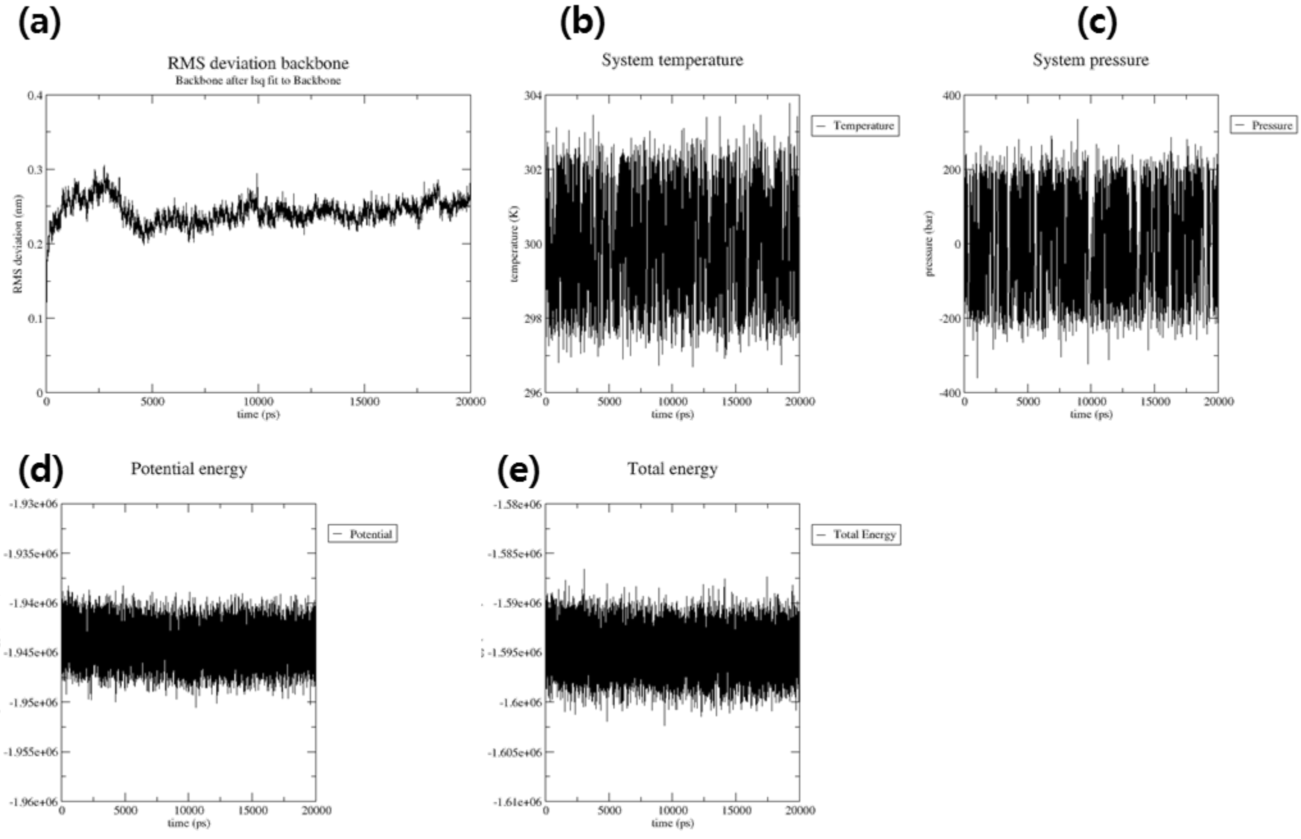

Supplementary Fig. 5. Analysis of the MD simulation of solvated *Zm*ASCH:RNA.
(a) Root mean square deviation of the protein backbone, (b) Temperature, (c) Pressure, (d) Potential energy, and (e) Total energy.

(a)

egend for links to other resources: UniGene GEO Gene Structure Map Viewer

Accession	
WP_011240781.1	DNA-binding protein [Zymomonas mobilis]
WP_037003891.1	Cro/CI family transcriptional regulator [Pseudomonas pseudoalcalige
OAV70416.1	50S ribosomal protein L22/unknown domain fusion protein [Bacteroid
WP_013832911.1	Cro/CI family transcriptional regulator [Novosphingobium sp. PP1Y]
WP_028095813.1	Cro/CI family transcriptional regulator [Dongia sp. URHE0060]
WP_052496829.1	Cro/CI family transcriptional regulator [Agrobacterium tumefaciens]
WP_035564357.1	Cro/CI family transcriptional regulator [Halomonas sp. KO116]
WP_037144084.1	MULTISPECIES: Cro/CI family transcriptional regulator [Rhizobium]
KEC72896.1	transcriptional regulator [Rhizobium leguminosarum bv. phaseoli CC(
WP_065282355.1	Cro/CI family transcriptional regulator [Rhizobium leguminosarum]
WP_027553695.1	Cro/CI family transcriptional regulator [Bradyrhizobium sp. Cp5.3]
WP_042456274.1	Cro/CI family transcriptional regulator [Rhodovulum sulfidophilum]
WP_042088088.1	Cro/CI family transcriptional regulator [alpha proteobacterium Q-1]
AAL52407.1	transcriptional regulator [Brucella melitensis bv. 1 str. 16M]
WP_002963867.1	MULTISPECIES: Cro/CI family transcriptional regulator [Brucella]
OAH16381.1	50S ribosomal protein L22/unknown domain fusion protein [Streptomy
WP_004688199.1	Cro/CI family transcriptional regulator [Brucella neotomae]
WP_026363906.1	MULTISPECIES: Cro/CI family transcriptional regulator [Agrobacteriur

WP_011240781	1	--------------------------------------MTdIPD-RKEAVISLWPEFAKAIVSGKKTVEFRRRIP-LPAL	40
WP_037003891	77	RIEAAFNADSEISTRLRE-ELALEEHSDRQLVLPIFSSDEmFLEESKDLILSIHPQYSDKILAGKKTVELRRRFPLATAK	155
OAV70416	1	--------------------------------------MEkISNVKKNLFISVKPEFAKKIIRKEKRIELRKVKP-HVEI	41
WP_013832911	78	RVNACFPADAPISLKLCD-TFDIEHGNDDQLVLPILGDDP--FRPQRDVVLSIKPKYSKSILDGRKTVELRRRFPVSAPN	154
WP_028095813	78	RIEATFRPEAMVKHGIAG-AFDAETLDDNQLVFPIFGDD--LFKPQRDVILSIRPQYSEKIMHGEKTVELRRRFPLSAPQ	154
WP_052496829	78	RVDAVFPADARLVLRVKE-TFDIEVGNDDQLVFPLLADEP--FRPQRDVVLSIRPQYSEKIMEGRKTVELRRRFPVSAPG	154
WP_035564357	78	NIHADFSLDA---INFRE-----EVTNERQLMLPIFNEGS--IESKRDIVISIRPEYSERILEGKKTVELRRRFPADSPQ	147
WP_037144084	78	RVEAVFPSDARLVLRIKE-AFDAEVGNDDQLVFPLLADEP--FRAQKDVVLSIRPQYSEKILEGRKTVELRRRFPISAPG	154
KEC72896	80	RVEAVFPSDARLVLRIKE-AFDAEVGNDDQLVFPLLADEP--FRAQKDVVLSIRPQYSEKILEGRKTVELRRRFPISAPG	156
WP_065282355	78	RVDAVFPADARLVLRIKE-AFDAEVGNDDQLVFPLLADEP--FRAQRDVVLSIRPQYSEKILEGRKTVELRRRFPVSAPG	154
WP_027553695	78	RIEATFTREALKKLRFEG-AFDPDLQDDDQFVFPIFGDD--LFRPKRDVVLSVRPVYSQKIMAGEKTVELRRRFPISAPS	154
WP_042456274	79	QVSAAFDAGSSLCLRVRE-AFDVELDQEDQLVLPIFAEEQ--FRPHRDVVLSIRPKFSSRIMSGEKTVELRRKFPAAAPE	155
WP_042088088	78	RLNAHFPVDTPIAARLQSvTFDIEQGHDDQLILPILADEP--FKPQRDVVLSIKPVYSEKILTGEKTVELRRRFPVAAPH	155
AAL52407	56	RIDAAFPVHSNLGVRIQA-DLGGLVADDEQYVLPIFGDGDgARPTKRDLVISIKPNYSGKIFDGVKTIELRRRFPLSIAA	134
WP_002963867	77	RIDAAFPVHSNLGVRIQA-DLGGLVADDEQYVLPIFGDGDgARPTKRDLVISIKPNYSGKIFDGVKTIELRRRFPLSIAA	155
OAH16381	1	---MNEHERSLLMSLHPRYASAILDGRKSVELRRQRV-AVPP	38
WP_004688199	77	RIDAAFPVHSNLGVRIQA-DLGGLVADDEQYVLPIFGDGDgARPTKRDLVISIKPNYSGKIFDGIKTIELRRRFPLSIAA	155
WP_026363906	77	RIDAAFPVHSEFGVRIQA-ELGGAVADDDQYVLPIFGDRDdARPAKRDIVISIKPNYSGKIFDGVKTIELRRRFPLSVSP	155
WP_037109289	77	RIDAAFPVHSELGVRIQA-ELGGAVADDDQYVLPIFGDRDdARPAKRDIVISIKPNYSGKIFDGVKTIELRRRFPLSVSP	155
WP_032451071	77	RIDAAFPVHSNLGVRIQA-DLGGLVADDEQYVLPIFGDGDgARPTKRDLVISIKPNYSGKIFDGVKTIELRRRFPLSIAA	155
WP_011517805	1	--MSTSGHVLISLEERHALNIFAGSKQVELRRRTM-HVEP	37
WP_046951222	1	--------------------------------------MAkTKN-RRVVFLPIKPEFAHKIINGKKNIEFRKKFS-SQEV	40
WP_053522499	1	--------------------------------------MAkTKN-RRVVFLPIKPEFAHKIINGEKNIEFRKKFS-SQEV	40
WP_035989018	1	--MLLDEHILISLERRHADHILDGSKCVELRRRPM-NVPV	37

WP_011240781	41	SARIWIYATRPVKSVIGFAYLEAIVQGDVNTLWSRYGREAFLSEQQYRDYFEGTEKATAFLLRDHQPIRA----INLDQL	116
WP_037003891	156	GTKVYIYSTSPVRAIVGSAEIAGIIKLPIKDMWKKYSKCAFIKKQAFESYFEGLSEGFALELKNAQAFDK---PIELLEL	232
OAV70416	42	GDVVIIYASSPLKSVIGFGKIQQIIEMSPEKMWKQYSSLLGIDKLRFDNYVNGKEKAVGIKIKEIQQIN----PIHLEAL	117
WP_013832911	155	GTLAVIYSTSPVKAMVGTAAIRDVLKLPIEQLWAEFEFAASIERDQFNAYFEGLEHGFALILDDAKAFSR---PLPLNEL	231
WP_028095813	155	GTIAYIYCTSPVRAIVGSAQISDVVKLPVEEIWKKYRNSAAIGKAEFDSYFDGLHKGFALKFANVRPLPR---KVDLSEL	231
WP_052496829	155	GTLAVIYSTSPIRAMVGIAEIKDVLKLPVQQIWSEFEDTAFIERNDFESYFQGLEFGFALLFEDVKPFSR---PVPLTEL	231
WP_035564357	148	NMVAYIYCTSPTMAMVGCVVVSKVIKLPINEIWEKYASVAFIKKEDFVKYFSGLDEGYVLELKHVKPLSR---QVGLKEL	224
WP_037144084	155	GTIAYIYSTSPVRAMVGVAEIKDVLKLPIEQIWAEFEDTAFIERADFDSYFQGVDFGFALLFEDVKSFAR---PIPLNEL	231
KEC72896	157	GTIAYIYSTSPVRAMVGVAEIKDVLKLPIEQIWAEFEDTAFIERADFDSYFQGVDFGFALLFEDVKSFAR---PIPLNEL	233
WP_065282355	155	GTIAYIYSTSPVRAMVGVAEIKDVLKLPIEQIWAEFEDTAFIERADFDSYFQGLDFGFALLFEDVKSFSR---PIPLNEL	231
WP_027553695	155	GTVAYIYSTSPVRAMVGSAEIENVVKMPIADIWKKFGSKARITRDDFESYFSGLEAGFALKFKNARAFAR---PIDLSEL	231
WP_042456274	156	GTIAYIYSTSPERAMVGVAEISSVRKLPIDEIWRRYADVAFIERPEFDRYFEGLHEGFALEFANVRPFET---PLGLAYL	232
WP_042088088	156	GALAYIYSSSPVKAMVGTASIRDVLKLPVEQIWNKFESKAFIERPLFDKYFEGLDVGVALVFDDVKSFTR---PLPLHEL	232
AAL52407	135	GATAYIYSTSPEMALVGTIKIENVERLQLRLLWKKHGQSASIKKADFDDYFSGLEEGFALKLSTPRRFTR---PLTLPEL	211

WP_002963867	156	GATAYIYSTSPEMALVGTIKIENVERLQLRLLWKKHGQSASIKKADFDDYFSGLEEGFALKLSTPRRFTR---PLTLPEL	232
OAH16381	39	GTKVILYATSPVMALVGTATVTAVQVGTPSVIWKAHKEHGAISRRDYLAYMEGAEQASALLLDAARPFSD---PIPLAHL	115
WP_004688199	156	GATAYIYSTSPEMALVGTIKIENVERLQLRLLWKKHGQSASIKKADFDDYFSGLEEGFALKLSTPRRFTR---PLTLPEL	232
WP_026363906	156	GATAYIYSTSPEMALVGAIKIENVERLELSALWKKHGRSASIKKADFDEYFSGLEEGFALKLSTPRRFAR---PLTLPEL	232
WP_037109289	156	GATAYVYSTSPEMALVGAIKIENVERLELSVLWKKHGRSASIKKADFDEYFSGLEEGFALKLATPRRFTR---PLTLPEL	232
WP_032451071	156	GATAYIYSTSPEMALVGTIKIENVERLQLRLLWKKHGQSASIKKADFDDYFXXXEEGFALKLSTPRRFTR---PLTLPEL	232
WP_011517805	38	GTVVWIYVKQPVGCVVGHAVVKATHSLSPRQVWGKFGSRSGLRRQEFFAYFEDVARAFVLELRDARRLEE---AITLETL	114
WP_046951222	41	-ETIVIYSSSPEKRVIGYATVDSIVIDTPDSLWKRFYKKGGIDKDRFSSYFNGKETGVGIRIKNVSRLKEavtPTQLGIQ	119
WP_053522499	41	-ETIVIYSSSPEKRVIGYATVDSIVIDTPNSLWKRFYKKGGIDKDRFSTYFNGKETGVGIRIKNVSRLKEavtPTQLGIQ	119
WP_035989018	38	GTTVWMYAKLPVASIVGRARVSGAHSLAPSTLWRRFAGVSGLTHGEFFSYFDGVSRGFALALENAERLPA---SVSLAAL	114

WP_011240781	117	KEiRANFQPPQSLTWLRKE--ETQKL-VSLTSQVE-	148
WP_037003891	233	RE-RFNFTPPQSFIYAKQE--MRRAL-MDEQTSLSN	264
OAV70416	118	RNiVPNFQPPQVYRYVSKE--MCNII-IDKDFHIYV[27]	177
WP_013832911	232	RE-KFGFEPPQSFLYAKRD--LRKAL-RDEPAIVSH	263
WP_028095813	232	RE-RFGFEPPQSFLYASPL--LRRAL-EHEFADIPN	263
WP_052496829	232	RE-KFGFEPPQSFLYASRD--LRKAL-RDEATVVSH	263
WP_035564357	225	RE-RFDFTPPQSFFYAKPK--LLKAL-QDEYSNVSH	256
WP_037144084	232	RE-RFSFEPPQSYLYASRD--LRKAL-RHEAAVVSH	263
KEC72896	234	RE-RFSFEPPQSYLYASRD--LRKAL-RHEAAVVSH	265
WP_065282355	232	RE-RFSFEPPQSYLYASRD--LRKAL-RHEAAVVSH	263
WP_027553695	232	RR-RFRFEPPQSFLYASPV--LRTAL-EDEYSDVSH	263
WP_042456274	233	RE-RFGFEPPQSFLYAKRD--LRKAL-KNEHAVVSH	264
WP_042088088	233	RE-KFGFEPPQSFLYAKHD--LRRAL-QDEPSSVSH	264
AAL52407	212	KE-RFGFKAPQSFLYAKPE--LQKAL-RNEHTNLPD	243
WP_002963867	233	KE-RFGFKAPQSFLYAKPE--LQKAL-RNEHTNLPD	264
OAH16381	116	RT-GGSFHPPQSYRYVDPD--TLRDW-VQGHPTAEE[42]	189
WP_004688199	233	KE-RFGFKAPQSFLYAKPE--LQKAL-RNEHTNLPD	264
WP_026363906	233	KE-RFGFKAPQSFLYAKPD--LQKAL-RHEHTNISD	264
WP_037109289	233	KE-RFGFKAPQSFLYAKPD--LQKAL-RNEHTNISD	264
WP_032451071	233	KE-RFGFKAPQSFLYAKPE--LQKAL-RNEHTNLPD	264
WP_011517805	115	RAvSSSFHPPQFFTRLTPEtaLARVLaRSKFSTLAQ[2]	152
WP_046951222	120	GAiPQNFKFLEK-GIITKL--ERNLI----------	142
WP_053522499	120	GAiPQNFKFLEK-GIITKL--ERNHI----------	142
WP_035989018	115	RDvSPGFQPPQFFMRLDGEgaLVRAM-SVHEVQFEE[10]	159

(b)

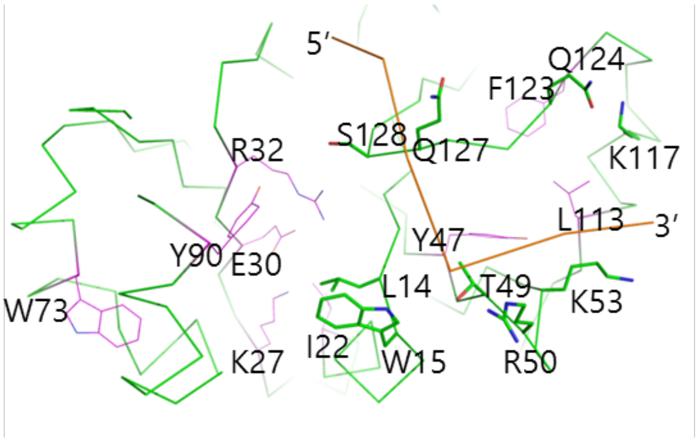

Supplementary Fig. 6. Expanded sequence alignment. (a) Identical residues are marked with red, while similar residues are marked with blue. (b) Spatial positions of the conserved residues displayed on the *Zm*ASCH structure (green ribbon). The simulated RNA molecule is displayed with an orange ribbon and residues are with thin and thick stick models, respectively.

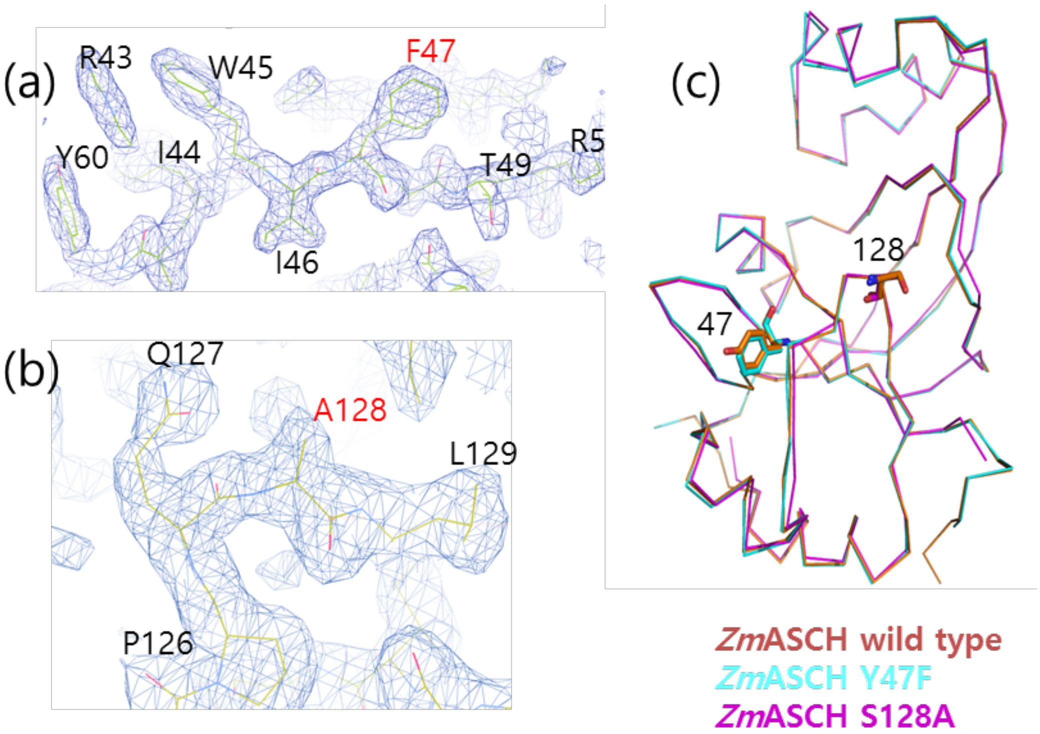

Supplementary Fig. 7. Structures of *Zm*ASCH variants, (a) *Zm*ASCH Y47F and (b) *Zm*ASCH S128A. Regions including the mutated sites, indicated with red, were displayed with stick models in the respective 2Fo-Fc electron density contoured at 1.5 σ for the Y47F at 2.0 Å resolution and 1.6 σ for the S128A at 2.4 Å resolution. (c) The crystal structures of the wild-type (orange), the Y47F mutant (cyan) and S128A mutant protein (magenta) were superposed and shown as Cα traces with residues of stick models. Coordinates for mutant proteins have been deposited under the PDB IDs of 5Y6C (Y47F) and 5Y6B (S128A).

Supplementary Table S2. Data Collection and Structure Refinement Statistics of *Zm*ASCH mutants.

**Data Collection**	**Y47F mutant**	**S128A mutant**
Space group	$P2_1$	$P3_121$
Unit cell dimensions		
a, b, c (Å),<br>α, β, γ (°)	53.13, 65.96, 91.86,<br>90.0, 90.2, 90.0	52.15, 52.15, 207.46,<br>90, 90, 120
Wavelength (Å)	0.9794	0.9794
Resolution (Å)	20-2.0 (2.03-2.00) [a]	20-2.4 (2.44-2.40) [a]
R_{sym}	6.4 (41.8)	8.4 (12.5)
$I/\sigma(I)$	12.3 (1.7)	21.5 (15.0)
Completeness (%)	97.6 (97.4)	99.8 (99.7)
Redundancy	2.8 (2.6)	9.3 (9.0)
**Refinement**		
Resolution (Å)	19.8-2.0 (2.05-2.00)	19.8-2.4 (2.48-2.40)
No. of reflections	41831	13550
R_{work} / R_{free}	22.2 (32.0) / 25.4 (38.5)	20.6 (25.0) / 23.5 (31.7)
No. atoms		
protein / water	4691 / 357	2335 / 130
RMSD		
bond lengths (Å) / angles (º)	0.009 / 0.993	0.020 / 1.660
Average B-values ($Å^2$)		
protein / water	31.1 / 31.1	26.8 / 25.8
Ramachandran plot (%)		
favored / allowed / outliers	97.3 / 2.2 / 0.5	98.6 / 1.4 / 0

[a] The numbers in parentheses are the statistics from the highest resolution shell.
